# Effects of Nutritional Supplementation during Pregnancy on Early Adult Disease Risk: Follow Up of Offspring of Participants in a Randomised Controlled Trial Investigating Effects of Supplementation on Infant Birth Weight

**DOI:** 10.1371/journal.pone.0083371

**Published:** 2013-12-13

**Authors:** John Macleod, Lie Tang, F. D. Richard Hobbs, Brian Wharton, Roger Holder, Shakir Hussain, Linda Nichols, Paul Stewart, Penny Clark, Steve Luzio, Jeff Holly, George Davey Smith

**Affiliations:** 1 School of Social and Community Medicine, University of Bristol, Bristol, United Kingdom; 2 Department of Primary Care and General Practice, University of Birmingham, Birmingham, United Kingdom; 3 Department of Primary Care Health Sciences, University of Oxford, Oxford, United Kingdom; 4 Institutes of Child Health, University of London and University of Birmingham, London and Birmingham, United Kingdom; 5 Centre for Endocrinology, Diabetes and Metabolism, University of Birmingham, Birmingham, United Kingdom; 6 Department of Clinical Biochemistry, University Hospital Birmingham, Birmingham, United Kingdom; 7 College of Medicine, Swansea University, Swansea, United Kingdom; 8 School of Clinical Sciences, University of Bristol, Bristol, United Kingdom; The Ohio State Unversity, United States of America

## Abstract

**Background:**

Observational evidence suggests that improving fetal growth may improve adult health. Experimental evidence from nutritional supplementation trials undertaken amongst pregnant women in the less developed world does not show strong or consistent effects on adult disease risk and no trials from the more developed world have previously been reported.

**Objective:**

To test the hypothesis that nutritional supplementation during pregnancy influences offspring disease risk in adulthood

**Design:**

Clinical assessment of a range of established diseases risk markers in young adult offspring of 283 South Asian mothers who participated in two trials of nutritional supplementation during pregnancy (protein/energy/vitamins; energy/vitamins or vitamins only) at Sorrento Maternity Hospital in Birmingham UK either unselected or selected on the basis of nutritional status.

**Results:**

236 (83%) offspring were traced and 118 (50%) of these were assessed in clinic. Protein/energy/vitamins supplementation amongst undernourished mothers was associated with increased infant birthweight. Nutritional supplementation showed no strong association with any one of a comprehensive range of markers of adult disease risk and no consistent pattern of association with risk across markers in offspring of either unselected or undernourished mothers.

**Conclusions:**

We found no evidence that nutritional supplements given to pregnant women are an important influence on adult disease risk however our study lacked power to estimate small effects. Our findings do not provide support for a policy of nutritional supplementation for pregnant women as an effective means to improve adult health in more developed societies.

## Introduction

The nutritional “fetal origins” hypothesis holds that physiological adaptation to nutritional status during gestation has life-long consequences for health[1,2]. Experimental animal models support the plausibility of this suggestion[3,4]. Human evidence for the importance of fetal nutrition as an influence on adult health is based mainly on observational studies showing that smaller birth size (which may reflect poorer nutrition in utero) is associated with greater adult risk of cardiovascular and metabolic disease[5-7]. It is possible that this association is confounded by factors independently associated both with birth size, and with adult disease risk[8]. The most reliable solution to this problem of confounding is random allocation of exposure status within an experimental design. If fetal nutrition does influence adult health it is unclear whether improving the nutritional status of pregnant women generally, or those showing evidence of under-nutrition, will benefit the adult health of their offspring[9]. A further issue in this regard is that over-nutrition during fetal life may lead to increased risk of some cancers[10]. A small number of follow-up studies of children whose mothers participated in trials of nutritional supplementation during pregnancy conducted in the less developed world have yielded inconclusive evidence in relation to effects on cardiovascular and metabolic outcomes and no evidence in relation to effects on cancer risk[11-14]. We assessed a group of young adults whose mothers participated in a randomised controlled trial of nutritional supplementation during pregnancy[15,16]. The primary outcome assessed in this trial was infant birth weight. Amongst mothers showing evidence of under-nutrition, supplements given in later pregnancy were effective in increasing offspring birth weight. We investigated the effect of supplementation on physiological markers of disease risk of young adult offspring of unselected mothers, and mothers with evidence of under-nutrition.

## Subjects and Methods

### Ethics Statement

All participants provided written informed consent. All study procedures were approved by South Birmingham Local Research Ethics Committee (LREC no. 0273). 

Details of participants and procedures in the original unselected and selected trials of nutritional supplementation at the Sorrento maternity hospital in Birmingham have been published elsewhere[15-17].Briefly, all South Asian mothers booking for maternity care prior to 20 weeks gestation between April 1979 and June 1980 were invited to participate. 153 mothers (see Figure S1) booking between April and October 1979 were randomised to receive one, of three, nutritional supplementation regimes between booking and delivery. Supplementation regimes were vitamins only (vitamin C 30mg and iron 3mg in 369 ml of flavoured carbonated water); carbohydrate and vitamins (as vitamins plus 1146KJ, the contemporary recommended extra daily allowance in pregnancy, of carbohydrate as glucose again in 369 ml of flavoured carbonated water) and protein, carbohydrate and vitamins (as carbohydrate and vitamins in 246 ml of flavoured carbonated water but with 11% of the energy, the contemporary recommended ratio, from protein in the form of chocolate flavoured skimmed milk powder).130 mothers booking between November 1979 and June 1980 were all given vitamins (Orovite 7: vitamin A 0.75mg, thiamine 1.4mg, riboflavin 1,7mg, pyridoxine 2.0mg, nicotinamide 18mg, ascorbic acid 60mg, calciferol 2.5 µg daily delivered in sachets for dissolving in water) and monitored till 28 weeks gestation. At this point, 45 mothers showing evidence of under-nutrition (on the basis of incremental triceps skin-fold measurements less than or equal to 20 µm per week) were again randomised to one of the three, supplementation regimes. In this trial those randomised to vitamins only continued to receive Orovite 7; those randomised to carbohydrate and vitamins received Orovite 7 plus 1810 KJ daily (additional allowance to allow for shorter period of supplementation) of carbohydrate in the form glucose syrup and those randomised to protein, carbohydrate and vitamins received Orovite 7 plus 1810 KJ daily 90% of energy as carbohydrate (glucose syrup) and 10% as protein (chocolate flavoured skimmed milk powder). Mothers showing no evidence of under-nutrition continued to receive vitamins. No placebo arm was included in the trials both for ethical reasons and reflecting concerns that a placebo controlled study might bias recruitment. Mothers recruited knew that they would at least receive vitamin supplements. The aim of the original trials was to evaluate the effects of protein and carbohydrate supplementation on birth weight and in this regard a comparison with vitamins only was appropriate. 

Supplements were delivered in batches to the mothers’ home at five week intervals. Blinding of mothers to their trial allocation group was not attempted. Empty containers from the previous delivery were collected to assess apparent consumption. Maternal compliance was also checked by study midwives and dieticians and monitored through blood biochemistry. Analysis was by intention to treat.

Personal identifier information for women who participated in the trial and their offspring were extracted from archived trial and maternity records. Individuals were then traced through the NHS central register.

All living offspring for whom contact details were available were sent a letter describing the study and inviting them to attend for a clinical assessment at the Wellcome Trust Clinical Research Facility at the Queen Elizabeth Hospital in Birmingham. Non-responders were sent reminder letters. Home-visits and telephone calls were used to confirm contact details and receipt of the study invitation. Results of maternal tracing and offspring follow up in clinic are summarised in a modified CONSORT diagram (see Figure S1).

Participants attended the clinic in a fasting state; a baseline blood sample was obtained prior to administration of a standard glucose tolerance test. Further blood samples were obtained at 30 and 120 minutes after the glucose load. Fasting blood samples were used to assess blood lipids, glucose, glycosylated haemoglobin, C peptide, testosterone and Sex Hormone Binding Globulin according to standard methods. Insulin and glucose were also estimated on the 30 and 120 minutes post glucose samples according to standard methods. Insulin resistance and beta cell sensitivity was estimated using the HOMA method[18]. Sex Hormone Binding Globulin and testosterone have been shown to be associated with insulin resistance[19].

Insulin like growth factors IGF1 and IGFBP3, the levels of which have been associated with increased risk of some common cancers [20,21], were also measured on fasting samples according to standard methods.

Participants were fitted with a Spacelabs 90207 ambulatory blood pressure monitor that was collected after completion of 24 hours of measurement allowing estimation of mean systolic and diastolic blood pressure over the measurement period[22].

Participants provided a 24 hour urine sample that was tested for steroid metabolites using gas chromatography/ mass spectroscopy. Urinary cortisol:cortisone (F/E) ratio was taken as a marker of 11 B- hydroxysteroid dehydrogenase 2 activity in the kidney (with increased ratios suggesting the impaired activity associated with failure to inactivate cortisol to cortisone associated with hypertension), tetrahydrocortisol + 5 allo tetrahydrocortisol: tetrahydrocortisone (THF + 5a – THF/ THE) ratio was taken to be a marker of 11 B- hydroxysteroid dehydrogenase 1(increased ratios suggesting the impaired activity associated with adiposity)[23]. Participants also underwent whole body dual-energy x-ray absorptiometry (DEXA) scanning to allow assessment of total body fat. Height and weight were assessed using standard methods and body mass index was calculated as weight in kilograms divided by height in metres squared. All clinic staff undertaking assessments were blind to initial trial allocation. Information on birth weight was taken from trial records.

### Statistical methods 

We investigated the primary null hypothesis that nutritional supplementation during pregnancy has no influence on disease risk in adulthood. We had no strong a priori basis to expect an effect of a particular size in relation to any of the disease risk markers measured with a particular supplementation regime. In the original Sorrento studies the strongest effect on birth weight was seen amongst offspring of mothers selected on the basis of their apparent under-nutrition who received protein, carbohydrate and vitamin supplements as described above. Mean values of infant birth weight and all adult outcome variables along with their standard errors were calculated according to original trial allocation and were compared using analysis of covariance amongst participants assessed as adults. Birth weight according to trial allocation was also examined amongst the larger group of participants successfully traced as adults. The original Sorrento studies were randomised therefore an unadjusted intention to treat analysis was appropriate. In the original studies a stronger effect on birth weight amongst under-nourished mothers was seen in a per protocol analysis excluding apparently non-compliant mothers[16]. We also repeated our analysis amongst offspring of mothers deemed to be nutritionally at risk excluding offspring of apparently non-compliant mothers. Around half of those eligible were successfully followed up in adulthood (see Figure S1) and the potential confounding factors of age, sex and adult adiposity were not evenly distributed across intervention groups in the follow up sample. Accordingly we repeated analyses adjusting for age sex and adult adiposity. 

Not all individuals assessed as adults had full information on all measures. To increase power and investigate the possibility that this might have led to bias we used multiple imputation of missing adult data using the ICE routine in Stata 10[24]. All variables in the analysis were used in the imputation model. After imputation, analyses were carried out on 5 imputed data sets and the results combined appropriately using Rubin’s Rules[25]. Adult analyses were then undertaken both on those with complete case information on the variables and on a dataset where missing values were imputed to allow comparison of the two. Where residuals from the analysis of covariance were significantly non-normal (Shapiro-Wilk p<0.05) bootstrap methods were invoked.

One participant in Trial I was excluded from the analyses as they had been measured on only one of the adult disease risk markers (BMI). 

Anonymised study data are available on request from the corresponding author. 

All analyses were performed using Stata 10[26].

## Results

118 offspring of mothers participating in the original Sorrento trials were assessed in clinic between June 2002 and October 2004. Participant characteristics at follow up are given in table **1**. Assessed offspring of mothers in the second trial who showed no evidence of under-nutrition are not considered in the analyses presented here (n=33). Amongst the 85 participants included in the analysis 40 (47%) were female (table 1). Mean age at assessment was 22.9 years (SD 0.9) and participants were healthy in terms of standard risk markers (table 1). 

**Table 1:** Patient characteristics of adult children from the Sorrento studies of maternal nutritional supplementation (values are mean ± SD, unless stated otherwise).

	**Trial I (unselective supplementation in all mothers from week 18)**			**Trial II (selective supplementation in nutritionally at risk mothers from week 28**)		
**Trial Group**	**Protein, Carbohydrate and Vitamins^*1*^**	**Carbohydrate and vitamins^*2*^**	**Vitamins only^*3*^**	**Protein, Carbohydrate and Vitamins^*4*^**	**Carbohydrate and vitamins^*5*^**	**Vitamins only^*6*^**
**n**	21	23	21	8	4	8
**Gender, n (%)**						
**Male**	13 (62%)	9 (39%)	12 (57%)	5 (63%)	1 (25%)	5 (62%)
**Female**	8 (38%)	14 (61%)	9 (43%)	3 (38%)	3 (75%)	3 (38%)
**Age at follow-up (years)**	23.0 ± 1.1	22.9 ± 0.8	23.0 ± 1.0	22.6 ± 0.5	22.3 ± 0.3	22.9 ± 0.6
**Systolic BP (mmHg)**	115.8 ± 8.5	115.8 ± 6.5	113.6 ± 6.8	115.4 ± 7.4	111.3 ± 10.3	110.5 ± 7.5
**Diastolic BP (mmHg)**	66.5 ± 6.6	68.6 ± 4.8	67.8 ± 4.2	67.3 ± 7.6	63.0 ± 2.9	66.5 ± 5.8
**Fasting glucose (mmol/l)**	5.0 ± 0.5	4.7 ± 0.5	4.8 ± 0.4	4.9 ± 0.3	4.4 ± 0.4	4.9 ± 0.3
**Fasting insulin (pmol/l)**	45.2 ± 26.6	63.3 ± 48.0	52.2 ± 30.3	25.8 ± 17.4	14.0	49.5 ± 29.4
**Total cholesterol**	4.5 ± 0.7	4.4 ± 1.0	4.4 ± 0.7	4.1 ± 0.7	4.3 ± 1.0	4.8 ± 0.8
**BMI (kg/m^2^)**	24.6 ± 4.8	24.0 ± 4.6	22.4 ± 4.0	22.6 ± 2.3	23.3 ± 2.7	23.4 ± 3.1
**Body fat (%)**	28.1 ± 9.2	29.5 ± 6.6	27.3 ± 8.0	25.6 ± 7.4	35.7 ± 4.3	26.6 ± 6.8

^1^ 1146 KJ energy daily with 11% from protein, 89% from carbohydrate; 30mg vitamin C, 3 mg iron

^2^ As 1. but 100% of energy from carbohydrate

^3^ 30mg vitamin C, 3 mg iron only

^4^ 1810 KJ energy daily with 10% from protein, 90% from carbohydrate, Orovite 7 (vitamin A 0.75mg; thiamine 1.4mg; riboflavin 1.7mg; pyridoxine 2.0mg; nicotinamide18mg; ascorbic acid 60mg; calciferol 2.5μg).

^5^ As 4. but 100% of energy from carbohydrate

^6^ Orovite 7 only

Distribution of those assessed according to allocation in the original trial is given in table **2**. Table 2 also shows offspring birth weight according to trial allocation in the original trial, amongst the traced cohort and amongst those assessed as adults. There is no strong evidence of differences in birth weight according to supplementation regime amongst offspring of unselected mothers (ANOVA F_2,62_ =0.73, p=0.48) . Amongst children of under-nourished mothers those receiving protein-energy supplements have higher birth weights however in the group of these children assessed as adults this difference is no longer apparent and any difference between groups is small and imprecisely estimated.

**Table 2:** Trial Allocation and birthweight in the Sorrento studies of maternal nutritional supplementation (values are mean ± SEM).

			**Trial I (unselective supplementation in all mothers from week 18)**				**Trial II (selective supplementation in nutritionally at risk mothers from week 28**)			
**Trial Group**			**Protein, Carbohydrate and Vitamins^*1*^**	**Carbohydrate and vitamins^*2*^**	**Vitamins only^*3*^**	**p^7^**	**Protein, Carbohydrate and Vitamins^*4*^**	**Carbohydrate and vitamins^*5*^**	**Vitamins only^*6*^**	**p^7^**
	**Mean Birthweight kg**	**Original trial (n)**	3.01 ± 0.06 (47)	3.02 ± 0.07 (50)	3.06 ± 0.07 (45)	0.87	3.34 ± 0.14 (14)	2.95 ± 0.15 (17)	3.01 ± 0.07 (14)	0.09
		**Traced participants (n**)	2.97 ± 0.07 (40)	2.96 ± 0.07 (41)	3.07 ± 0.08 (38)	0.53	3.34 ± 0.14 (14)	2.96 ± 0.17 (15)	3.01 ± 0.07 (13)	0.15
		**Assessed in clinic (n)^*8*^**	3.11 ± 0.09 (21)	2.95 ± 0.08 (23)	3.08 ± 0.12 (21)	0.48	3.23 ± 0.13 (8)	3.42 ± 0.15 (4)	3.03 ± 0.11 (8)	0.18

^1^ 1146 KJ energy daily with 11% from protein, 89% from carbohydrate; 30mg vitamin C, 3 mg iron

^2^ As 1. but 100% of energy from carbohydrate

^3^ 30mg vitamin C, 3 mg iron only

^4^ 1810 KJ energy daily with 10% from protein, 90% from carbohydrate, Orovite 7 (vitamin A 0.75mg; thiamine 1.4mg; riboflavin 1.7mg; pyridoxine 2.0mg; nicotinamide18mg; ascorbic acid 60mg; calciferol 2.5μg).

^5^ As 4. but 100% of energy from carbohydrate

^6^ Orovite 7 only

^7^ p-value for differences in mean birthweight between trial arms (from analysis of variance)

^8^ Analyses of variance to compare birth weights of those assessed in clinic and those in original trial but not assessed in clinic were not significant for either trial. Trial I: F_1,137_=0.14, p=0.71, Trial II: F_1,43_=1.42, p=0.24

Table **3** shows offspring cardiovascular and metabolic disease risk according to trial allocation amongst children assessed in clinic whose mothers received nutritional supplements from week 18 of gestation and were unselected on the basis of nutritional status. There is no consistent evidence of differences in risk status according to supplementation regime either before or after multiple imputation of missing values or adjustment for age, gender and adult adiposity. Fasting glucose is higher amongst offspring of mothers receiving protein energy supplements though this effect is only apparent in the unadjusted analysis and is in the opposite direction to effects on fasting insulin which is lowest amongst offspring of mothers receiving protein energy supplements (an effect most apparent after adjustment). 

**Table 3:** Adult disease risk markers amongst offspring of mothers participating in the Sorrento studies of maternal nutritional supplementation who were assessed as adults before and after imputation of missing values - Trial I (supplementation in all mothers after 18 weeks).

Risk marker	Trial Group^*^	**Protein, Carbohydrate and Vitamins**	**Carbohydrate and vitamins**	**Vitamins only**	**P^1^**		**P^2^**
		Mean ± SEM^**^ (n)	**Mean ± SEM (n)**	**Mean ± SEM (n)**			
Blood pressure	**Mean 24Hr Systolic BP (mmHg)**	115.8 ± 2.1 (16)	115.8 ± 1.4 (22)	113.6 ± 1.6 (19)	0.57		0.57
	With imputation^***^	115.3 ± 2.0 (21)	115.8 ± 1.4 (22)	113.8 ± 1.7 (21)	0.69		0.47
	Excluding non compliant mothers****	115.5 ± 2.5 (13)	115.8 ± 1.5 (20)	113.4 ± 1.9 (15)	0.64		0.69
	**Mean 24Hr Diastolic BP (mmHg)**	66.5 ± 1.6 (16)	68.6 ± 1.0 (22)	67.8 ± 1.0 (19)	0.46		0.43^3^
	*With imputation*	66.3 ± 1.4 (21)	68.6 ± 1.0 (22)	67.6 ± 1.0 (21)	0.35^3^		0.31^3^
	*Excluding non compliant mothers*	68.0 ± 1.8 (13)	68.6 ± 1.1 (20)	67.7 ± 1.2 (15)	0.86		0.88
**Markers of glucose tolerance and insulin resistance**	**Fasting glucose (mmol/l)^*4*^**	5.0 ± 0.1 (21)	4.7 ± 0.1 (22)	4.8 ± 0.1 (21)	0.05^3^		0.62
	*Excluding non compliant mothers*	5.1 ± 0.1 (16)	4.7 ± 0.1 (20)	4.7 ± 0.1 (16)	0.002^3^		0.29
	**30 mins post load glucose (mmol/l)**	7.9 ± 0.3 (21)	7.6 ± 0.3 (21)	7.7 ± 0.3 (21)	0.83		0.71
	*With imputation*	7.9 ± 0.3 (21)	7.6 ± 0.3 (22)	7.7 ± 0.3 (21)	0.73		0.84
	*Excluding non compliant mothers*	7.8 ± 0.4 (16)	7.6 ± 0.3 (20)	7.7 ± 0.4 (16)	0.91		0.85
	**120 mins post load glucose (mmol/l)**	5.2 ± 0.3 (20)	5.3 ± 0.2 (21)	4.8 ± 0.2 (21)	0.34		0.69
	*With imputation*	5.2 ± 0.3 (21)	5.2 ± 0.2 (22)	4.8 ± 0.2 (21)	0.46		0.84
	*Excluding non compliant mothers*	5.4 ± 0.4 (16)	5.2 ± 0.2 (20)	4.8 ± 0.2 (16)	0,.41		0.46
	**Fasting insulin (pmol/l)**	45.2 ± 6.3 (18)	63.3 ± 12.0 (16)	52.2 ± 8.4 (13)	0.38^3^		0.03
	*With imputation*	44.3 ± 7.1 (21)	57.1 ± 10.9 (22)	51.8 ± 8.6 (21)	0.69^3^		0.22^3^
	*Excluding non compliant mothers*	46.8 ± 7.9 (14)	62.3 ± 12.8 (15)	55.2 ± 9.5 (10)	0.58^3^		0.24
	**30 mins post load insulin (pmol/l)**	429.1 ± 58.8 (16)	515.4 ± 62.0 (19)	488.2 ± 68.7 (17)	0.65^3^		0.54
	*With imputation*	436.7 ± 68.9 (21)	510.0 ± 62,9 (22)	479.0 ± 77.5 (21)	0.82^3^		0.74^3^
	*Excluding non compliant mothers*	407.7 ± 67.5 (13)	513.1 ± 62.0 (17)	486.8 ± 80.5 (13)	0.58^3^		0.72
	**120 mins post load insulin (pmol/l)**	400.7 ± 119.6 (14)	409.1 ± 85.3 (16)	317.9 ± 59.6 (12)	0.79^3^		0.93
	*With imputation*	393.8 ± 101.2 (21)	365.5 ± 77.1 (22)	294.9 ± 53.8 (21)	0.72^3^		0.98^3^
	*Excluding non compliant mothers*	449.6 ± 148.6 (11)	412.5 ± 91.1 (15)	311.1 ± 74.6 (9)	0.74^3^		0.85
	**HbA1c (%)**	5.02 ± 0.09 (20)	4.93 ± 0.08 (22)	5.03 ± 0.11 (20)	0.75^3^		0.67^3^
	*With imputation*	5.02 ± 0.09 (21)	4.93 ± 0.08 (22)	5.01 ± 0.11 (21)	0.78		0.78^3^
	*Excluding non compliant mothers*	4.99 ± 0.10 (15)	5.00 ± 0.07 (20)	4.93 ± 0.14 (15)	0.88^3^		0.49^3^
	**Cpeptide fasting (nmol/l)**	0.56 ± 0.05 (18)	0.60 ± 0.07 (22)	0.58 ± 0.06 (19)	0.89^3^		0.06
	*With imputation*	0.58 ± 0.07 (21)	0.60 ± 0.07 (22)	0.60 ± 0.06 (21)	0.95^3^		0.50^3^
	*Excluding non compliant mothers*	0.58 ± 0.07 (14)	0.60 ± 0.08 (20)	0.57 ± 0.07 (15)	0.93^3^		0.22
	**B cell function (%)**	86.5 ± 8.4 (18)	109.7 ± 14.4 (16)	98.2 ± 10.1 (13)	0.32^3^		0.05
	*With imputation*	83.7 ± 9.2 (21)	104.8 ± 13.0 (22)	97.8 ± 10.7 (21)	0.48^3^		0.15^3^
	*Excluding non compliant mothers*	85.9 ± 10.4 (14)	109.3 ± 15.4 (15)	106.1 ± 11.3 (10)	0.43^3^		0.23
	**Insulin sensitivity (%)**	158.2 ± 18.2 (18)	173.3 ± 43.1 (16)	140.4 ± 21.1 (13)	0.75^3^		0.49^3^
	*With imputation*	166.3 ± 25.7 (21)	194.6 ± 40.8 (22)	159.3 ± 24.9 (21)	0.71^3^		0.45^3^
	*Excluding non compliant mothers*	159.8 ± 22.1 (14)	180.4 ± 45.5 (15)	126.8 ± 20.4 (10)	0.64^3^		0.67^3^
	**Insulin resistance**	0.84 ± 0.12 (18)	1.16 ± 0.22 (16)	0.97 ± 0.15 (13)	0.41^3^		0.03
	*With imputation*	0.83 ± 0.13 (21)	1.04 ± 0.20 (22)	0.95 ± 0.16 (21)	0.72^3^		0.24^3^
	*Excluding non compliant mothers*	0.87 ± 0.15 (14)	1.14 ± 0.23 (15)	1.01 ± 0.17 (10)	0.60^3^		0.26
Serum lipids	**Total cholesterol (mmol/l)**	4.52 ± 0.16 (21)	4.43 ± 0.21 (21)	4.38 ± 0.16 (21)	0.84^3^		0.97
	*With imputation*	4.52 ± 0.16 (21)	4.43 ± 0.21 (22)	4.38 ± 0.16 (21)	0.83^3^		0.91
	*Excluding non compliant mothers*	4.59 ± 0.20 (16)	4.32 ± 0.21 (19)	4.48 ± 0.19 (16)	0.63^3^		0.70
	**HDL cholesterol (mmol/l)**	1.37 ± 0.07 (20)	1.39 ± 0.08 (20)	1.33 ± 0.08 (20)	0.85^3^		0.52^3^
	*With imputation*	1.37 ± 0.07 (21)	1.36 ± 0.08 (22)	1.33 ± 0.09 (21)	0.94^3^		0.76^3^
	*Excluding non compliant mothers*	1.35 ± 0.07 (16)	1.40 ± 0.08 (18)	1.35 ± 0.10 (16)	0.87^3^		0.70^3^
	**Triglycerides (mmol/l)**	1.03 ± 0.08 (21)	0.86 ± 0.07 (21)	1.06 ± 0.15 (21)	0.41^3^		0.33^3^
	*With imputation*	1.03 ± 0.08 (21)	0.85 ± 0.07 (22)	1.06 ± 0.15 (21)	0.32^3^		0.27^3^
	*Excluding non compliant mothers*	0.98 ± 0.08 (16)	0.83 ± 0.07 (19)	1.15 ± 0.19 (16)	0.15^3^		0.05^3^
Insulin like growth factors	**IGF1 (ng/ml**)	168.2 ± 17.7 (18)	171.0 ± 9.7 (22)	184.4 ± 13.1 (19)	0.67^3^		0.61
	*With imputation*	172.2 ± 16.7 (21)	171.0 ± 9.7 (22)	185.7 ± 15.2 (21)	0.75^3^		0.89^3^
	*Excluding non compliant mothers*	174.7 ± 19.4 (14)	172.5 ± 10.6 (20)	192.2 ± 15.5 (15)	0.60^3^		0.31
	**IGFBP3**	4327 ± 232 (18)	4363 ± 123 (22)	4211 ± 203 (19)	0.83		0.66
	*With imputation*	4327 ± 238 (21)	4363 ± 123 (22)	4221 ± 190 (21)	0.86		0.69
	*Excluding non compliant mothers*	4410 ± 273 (14)	4319 ± 132 (20)	4376 ± 216 (15)	0.95		0.98
Adiposity	**Body Mass Index^*4*^**	24.6 ± 1.0 (21)	24.0 ± 1.0 (23)	22.4 ± 0.9 (21)	0.25^3^		0.22
	*Excluding non compliant mothers*	24.0 ± 1.0 (16)	24.3 ± 1.0 (20)	22.1 ± 1.1 (16)	0.30^3^		0.32
	**Dexa_Total_Fat (%)**	28.1 ± 2.4 (15)	29.6 ± 1.5 (19)	27.3 ± 2.0 (16)	0.69		0.49
	*With imputation*	28.1 ± 2.0 (21)	30.3 ± 1.4 (22)	27.0 ± 1.9 (21)	0.40		0.77
	*Excluding non compliant mothers*	26.3 ± 2.4 (12)	30.0 ± 1.7 (17)	27.3 ± 2.5 (12)	0.43		0.29
Urinary steroid ratios	**Urinary F/E ratio**	0.79 ± 0.06 (18)	0.81 ± 0.04 (16)	0.70 ± 0.03 (14)	0.31		0.31^3^
	*With imputation*	0.78 ± 0.06 (21)	0.79 ± 0.04 (22)	0.70 ± 0.04 (21)		0.24	0.38^3^
	*Excluding non compliant mothers*	0.78 ± 0.07 (14)	0.81 ± 0.04 (15)	0.71 ± 0.04 (11)		0.45	0.36^3^
	**Urinary THF + 5aTHF/THE ratio**	0.94 ± 0.06 (18)	0.83 ± 0.04 (16)	0.88 ± 0.06 (14)		0.45^3^	0.57
	*With imputation*	0.93 ± 0.06 (21)	0.88 ± 0.05 (22)	0.88 ± 0.06 (21)		0.81^3^	0.74^3^
	*Excluding non compliant mothers*	0.90 ± 0.06 (14)	0.83 ± 0.04 (15)	0.92 ± 0.07 (11)		0.52^3^	0.57
	**Testosterone – Males (nmol/l)**	18.2 ± 1.4 (12)	15.5 ± 2.2 (8)	16.9 ± 1.8 (10)		0.58	0.18
	*With imputation*	18.2 ± 1.4 (13)	15.4 ± 2.2 (9)	16.9 ± 1.8 (12)		0.56	0.51
	*Excluding non compliant mothers*	18.4 ± 1.9 (9)	16.2 ± 2.4 (7)	19.1 ± 2.0 (7)		0.61	0.47
	**Testosterone – Females (nmol/l)**	1.9 ± 0.4 (5)	1.4 ± 0.1 (13)	1.4 ± 0.2 (9)		0.24	0.36
	*With imputation*	2.0 ± 0.3 (8)	1.4 ± 0.1 (13)	1.4 ± 0.2 (9)		0.04	0.28
	*Excluding non compliant mothers*	1.9 ± 0.4 (5)	1.5 ± 0.1 (12)	1.5 ± 0.2 (8)		0.33	0.47
	**SHBG – Males (nmol/l)**	17.8 ± 1.4 (12)	15.6 ± 1.7 (9)	20.2 ± 3.5 (10)		0.45^3^	0.37
	*With imputation*	18.2 ± 1.9 (13)	15.6 ± 1.7 (9)	19.9 ± 3.3 (12)		0.55^3^	0.81
	*Excluding non compliant mothers*	17.1 ± 1.8 (9)	16.8 ± 1.3 (8)	18.8 ± 3.2 (7)		0.80	0.62
	**SHBG – Females (nmol/l)**	47.5 ± 18.4 (6)	56.8 ± 17.1 (13)	47.7 ± 9.4 (9)		0.90^3^	0.86^3^
	*With imputation*	45.5 ± 16.7 (8)	56.8 ± 17.1 (13)	47.7 ± 9.4 (9)		0.85^3^	0.53^3^
	*Excluding non compliant mothers*	52.7 ± 21.6 (5)	55.7 ± 18.6 (12)	53.0 ± 8.7 (8)		0.98^3^	0.99^3^

^1^ p value for difference between groups

^2^ p value for difference between groups adjusted for BMI, Dexa, age and gender (BMI or Dexa fat are omitted as covariates when they are the dependent)

^3^ p value has been derived by bootstrapping because residuals from ANOVA were not normally distributed (Shapiro-Wilk test found to be significant).

^4^ Imputation not required.

* Trial Group” refers to original experimental treatment allocation; **Standard error of the mean; ***Multiple imputation of missing values as described in text; ****Per protocol analysis excluding mothers with evidence for non-compliance as described in text

Table **4** shows between group differences in risk factors amongst offspring of unselected mothers with the vitamins only group as the reference category. There is no strong evidence apparent of differences in individual risk factors between groups and no evidence of a pattern of lower risk in either of the energy supplementation groups compared to the vitamins only group. 

**Table 4:** Adult disease risk markers amongst offspring of mothers participating in the Sorrento studies of maternal nutritional supplementation who were assessed as adults: between group differences^1^ - Trial I (supplementation in all mothers after 18 weeks).

**Risk marker**			**Protein, Carbohydrate and Vitamins**		**Carbohydrate and vitamins**	
			**Difference**	**(95% CI)**	**Difference**	**(95% CI)**
**Blood pressure**	**Mean 24hr Systolic BP (mmHg)**	Unadjusted	2.12	(-2.78 to 7.01)	2.19	(-2.33 to 6.71)
		Adjusted^*^	0.04	(-5.48 to 5.56)	2.28	(-2.64 to 7.20)
	**Mean 24hr Diastolic BP (mmHg)**	Unadjusted	-1.34	(-4.87 to 2.19)	0.79	(-2.47 to 4.05)
		Adjusted**^*2*^**	-2.04	(-5.97 to 1.89)	0.25	(-3.28 to 3.77)
**Markers of glucose tolerance and insulin resistance**	**Fasting glucose (mmol/l)**	Unadjusted**^*2*^**	0.19	(-0.09 to 0.47)	-0.13	(-0.41 to 0.15)
		Adjusted	0.01	(-0.31 to 0.33)	-0.12	(-0.42 to 0.17)
	**30 mins post load glucose (mmol/l)**	Unadjusted	0.17	(-0.70 to 1.02)	-0.09	(-0.95 to 0.77)
		Adjusted	0.40	(-0.62 to 1.43)	0.08	(-0.89 to 1.04)
	**120 mins post load glucose (mmol/l)**	Unadjusted	0.44	(-0.29 to 1.18)	0.49	(-0.24 to 1.21)
		Adjusted	0.19	(-0.68 to 1.07)	0.35	(-0.47 to 1.17)
	**Fasting insulin (pmol/l)**	Unadjusted**^*2*^**	-6.82	(-32.11 to 18.46)	10.47	(-15.56 to 36.51)
		Adjusted	-12.17	(-33.88 to 9.55)	14.94	( -6.82 to 36.70)
	**30 mins post load insulin (pmol/l)**	Unadjusted**^*2*^**	-60.81	(-238.72 to 117.10)	30.30	(-139.75 to 200.34)
		Adjusted	-91.19	(-286.73 to 104.36)	2.27	(-178.68 to 183.21)
	**120 mins post load insulin (pmol/l)**	Unadjusted**^*2*^**	76.32	(-189.60 to 342.24)	93.14	(-164.68 to 350.96)
		Adjusted	47.35	(-221.98 to 316.68)	41.99	(-212.89 to 296.87)
	**HbA1c (%)**	Unadjusted**^*2*^**	-0.01	(-0.26 to 0.25)	-0.09	(-0.34 to 0.16)
		Adjusted**^*2*^**	-0.07	(-0.35 to 0.20)	0.03	(-0.22 to 0.28)
	**Cpeptide fasting (nmol/l)**	Unadjusted**^*2*^**	-0.03	(-0.20 to 0.15)	0.02	(-0.15 to 0.19)
		Adjusted	-0.12	(-0.26 to 0.02)	0.04	(-0.09 to 0.17)
	**B cell function (%)**	Unadjusted**^*2*^**	-11.44	(-42.56 to 19.68)	10.78	(-21.28 to 42.84)
		Adjusted	-14.12	(-43.55 to 15.31)	19.60	(-9.88 to 49.09)
	**Insulin sensitivity (%)**	Unadjusted**^*2*^**	17.86	(-63.52 to 99.24)	33.79	(-49.88 to 117.46)
		Adjusted**^*2*^**	29,.98	(-39.25 to 99.18)	7.84	(-61.44 to 77.12)
	**Insulin resistance**	Unadjusted**^*2*^**	-0.12	(-0.58 to 0.34)	0.18	(-0.29 to 0.66)
		Adjusted	-0.22	(-0.62 to 0.17)	0.26	(-0.13 to 0.66)
**Serum lipids**	**Total cholesterol (mmol/l)**	Unadjusted**^*2*^**	0.16	(-0.33 to 0.65)	0.07	(-0.42 to 0.56)
		Adjusted	-0.01	(-0.57 to 0.54)	0.05	(-0.47 to 0.57)
	**HDL cholesterol (mmol/l)**	Unadjusted**^*2*^**	0.05	(-0.17 to 0.26)	0.05	(-0.16 to 0.26)
		Adjusted**^*2*^**	0.14	(-0.11 to 0.39)	0.07	(-0.17 to 0.31)
	**Triglycerides (mmol/l)**	Unadjusted**^*2*^**	-0.03	(-0.32 to 0.26)	-0.20	(-0.49 to 0.09)
		Adjusted**^*2*^**	-0.21	(-0.54 to 0.11)	-0.25	(-0.56 to 0.06)
**Insulin like growth factors**	**IGF1 (ng/ml)**	Unadjusted**^*2*^**	-16.25	(-53.82 to 21.31)	-14.07	(-49.78 to 21.64)
		Adjusted	-15.28	(-51.34 to 20.78)	0.44	(-32.96 to 33.84)
	**IGFBP3**	Unadjusted	116.22	(-423.67 to 656.11)	152.41	(-361.66 to 666.48)
		Adjusted	90.27	(-452.30 to 632.83)	225.17	(-277.45 to 727.78)
**Adiposity**	**Body Mass index**	Unadjusted**^*2*^**	2.32	(-0.33 to 4.96)	1.68	(-0.94 to 4.31)
		Adjusted	1.60	(-0.32 to 3.51)	0.36	(-1.47 to 2.18)
	**Dexa total fat (%)**	Unadjusted	0.84	(-4.85 to 6,53)	2.28	(-3.09 to 7.66)
		Adjusted	-1.41	(-4.06 to 1.24)	-0.06	(-2.55 to 2.42)
**Urinary steroid ratios**	**Urinary F/E ratio**	Unadjusted	0.08	(-0.05 to 0.22)	0.10	(-0.04 to 0.24)
		Adjusted**^*2*^**	0.02	(-0.12 to 0.15)	0.11	(-0.03 to 0.25)
	**Urinary THF + 5aTHF/THE ratio**	Unadjusted**^*2*^**	0.06	(-0.09 to 0.21)	-0.05	(-0.20 to 0.11)
		Adjusted	0.04	(-0.15 to 0.23)	-0.06	(-0.25 to 0.13)
	**Testosterone – Males (nmol/l)**	Unadjusted	1.31	(-3.56 to 6.18)	-1.37	(-6.76 to 4.03)
		Adjusted	3.73	(-0.94 to 8.42)	-0.25	(-4.98 to 4.48)
	**Testosterone – Females (nmol/l)**	Unadjusted	0.46	(-0.19 to 1.10)	-0.04	(-0.54 to 0.46)
		Adjusted	0.25	(-0.43 to 0.92)	-0.17	(-0.72 to 0.38)
	**SHBG – Males (nmol/l)**	Unadjusted**^*2*^**	-2.38	(-8.48 to 3.72)	-4.66	(-11.29 to 1.98)
		Adjusted	-0.01	(-6.60 to 6.58)	-4.07	(-10.73 to 2.60)
	**SHBG – Females (nmol/l)**	Unadjusted**^*2*^**	-0.60	(-50.83 to 49.64)	9.14	(-31.01 to 49.30)
		Adjusted**^*2*^**	24.40	(-29.89 to 78.68)	14.22	(-29.41 to 57.84)

^1^ between group differences based on the complete case analysis and are shown with Vitamins only group as reference category

^2^ estimates have been bootstrapped as residuals were not normally distributed (Shapiro-Wilk test <0.05)

* adjusted for BMI, Dexa, age and gender (BMI or Dexa fat are omitted as covariates when they are the dependent)

Table **5** shows offspring cardiovascular and metabolic disease risk according to trial allocation amongst children assessed in clinic whose mothers received nutritional supplements from week 28 of gestation on the basis of their showing evidence of under-nutrition. Again, there is no consistent evidence of differences in risk status according to supplementation regime either before or after multiple imputation of missing values or adjustment for age, gender and adult adiposity. 

**Table 5:** Adult disease risk markers amongst offspring of mothers participating in the Sorrento studies of maternal nutritional supplementation who were assessed as adults before and after imputation of missing values - Trial II (supplementation in nutritionally at risk mothers after 28 weeks).

Risk marker	Trial Group^*^	**Protein, Carbohydrate and Vitamins**	**Carbohydrate and vitamins**	**Vitamins only**	**P^1^**	**P^2^**
		Mean ± SEM^**^ (n)	**Mean ± SEM (n)**	**Mean ± SEM (n)**		
Blood pressure	**Mean 24Hr Systolic BP (mmHg)**	115.4 ± 2.6 (8)	111.3 ± 5.1 (4)	110.5 ± 3.1 (6)	0.50	0.79
	With imputation^***^	115.4 ± 2.6 (8)	111.3 ± 5.1 (4)	111.2 ± 2.6 (8)	0.52	0.86
	*Excluding non compliant mothers* ^****^	116.4 ± 2.8 (7)	115.3 ± 4.4 (3)	110.5 ± 3.1 (6)	0.37	0.58
	**Mean 24Hr Diastolic BP (mmHg)**	67.3 ± 2.7 (8)	63.0 ± 1.5 (4)	66.5 ± 2.3 (6)	0.54	0.38
	*With imputation*	67.3 ± 2.7 (8)	63.0 ± 1.5 (4)	65.8 ± 2.3 (8)	0.53	0.34
	*Excluding non compliant mothers*	69.0 ± 2.3 (7)	64.0 ± 1.5 (3)	66.5 ± 2.3 (6)	0.43	0.48
**Markers of glucose tolerance and insulin resistance**	**Fasting glucose (mmol/l)^*3*^**	4.9 ± 0.1 (8)	4.4 ± 0.2 (4)	4.9 ± 0.1 (8)	0.01^4^	0.19
	*Excluding non compliant mothers*	4.9 ± 0.1 (7)	4.5 ± 0.3 (3)	4.9 ± 0.1 (8)	0.13^4^	0.34
	**30 mins post load glucose (mmol/l)^*3*^**	7.1 ± 0.5 (8)	6.8 ± 1.0 (4)	8.5 ± 0.6 (8)	0.14	0.21
	*Excluding non compliant mothers*	6.9 ± 0.5 (7)	7.0 ± 1.4 (3)	8.5 ± 0.6 (8)	0.18	0.24
	**120 mins post load glucose (mmol/l)^*3*^**	5.6 ± 0.4 (8)	5.1 ± 1.0 (4)	4.9 ± 0.4 (8)	0.53	0.30
	*Excluding non compliant mothers*	5.7 ± 0.5 (7)	5.5 ± 1.4 (3)	4.9 ± 0.4 (8)	0.51	0.46
	**Fasting insulin (pmol/l)**	25.8 ± 7.8 (5)	14.0 (1)	49.5 ± 12.0 (6)	0.24	0.21
	*With imputation*	30.3 ± 8.0 (8)	33.5 ± 21.7 (4)	44.0 ± 12.1 (8)	0.82^4^	0.62^3^
	*Excluding non compliant mothers*	22.0 ± 8.8 (4)	14.0 (1)	49.5 ± 12.0 (6)	0.23	0.21
	**30 mins post load insulin (pmol/l)**	392.8 ± 62.4 (6)	464.3 ± 293.5 (3)	523.0 ± 113.2 (6)	0.71^4^	0.99
	*With imputation*	421.3 ± 84.9 (8)	457.6 ± 221.9 (4)	503.8 ± 103.6 (8)	0.85^4^	0.96
	*Excluding non compliant mothers*	404.2 ± 75.2 (5)	620.0 ± 431.0 (2)	523.0 ± 113.2 (6)	0.65	0.99
	**120 mins post load insulin (pmol/l)**	410.3 ± 138.9 (6)	161.0 (1)	431.2 ± 177.2 (6)	0.85^4^	0.22
	*With imputation*	371.5 ± 114.7 (8)	423.9 ± 231.8 (4)	375.0 ± 143.0 (8)	0.97^4^	0.48
	*Excluding non compliant mothers*	468.8 ± 154.3 (5)	161.0 (1)	431.2 ± 177.2 (6)	0.78	0.22
	**HbA1c (%)^*3*^**	4.88 ± 0.08 (8)	4.85 ± 0.10 (4)	4.99 ± 0.14 (8)	0.69	0.68
	*Excluding non compliant mothers*	4.89 ± 0.10 (7)	4.90 ± 0.12 (3)	4.99 ± 0.14 (8)	0.82	0.80
	**Cpeptide fasting (nmol/l)^*3*^**	0.44 ± 0.05 (8)	0.42 ± 0.10 (4)	0.65 ± 0.12 (8)	0.20	0.06
	*Excluding non compliant mothers*	0.44 ± 0.06 (7)	0.47 ± 0.11 (3)	0.65 ± 0.12 (8)	0.31	0.07
	**B cell function (%)**	57.8 ± 14.5 (5)	50.4 (1)	90.9 ± 14.1 (6)	0.26	0.22
	*With imputation*	66.9 ± 13.5 (8)	81.9 ± 33.3 (4)	82.9 ± 16.0 (8)	0.80	0.89^3^
	*Excluding non compliant mothers*	51.6 ± 16.9 (4)	50.4 (1)	90.9 ± 14.1 (6)	0.22	0.22
	**Insulin sensitivity (%)**	342.5 ± 136.1 (5)	391.0 (1)	140.0 ± 29.2 (6)	0.18^4^	0.29
	*With imputation*	290.2 ± 94.0 (8)	280.1 ± 120.2 (4)	179.6 ± 62.2 (8)	0.60^4^	0.46^3^
	*Excluding non compliant mothers*	395.5 ± 161.8 (4)	391.0 (1)	140.0 ± 29.2 (6)	0.12^4^	0.29
	**Insulin resistance**	0.48 ± 0.14 (5)	0.26 (1)	0.92 ± 0.22 (6)	0.23	0.20
	With imputation	0.56 ± 0.15 (8)	0.61 ± 0.39 (4)	0.82 ± 0.22 (8)	0.81^4^	0.59^3^
	*Excluding non compliant mothers*	0.41 ± 0.16 (4)	0.26 (1)	0.92 ± 0.22 (6)	0.22	0.20
Serum lipids	**Total cholesterol (mmol/l)^*3*^**	4.11 ± 0.23 (8)	4.30 ± 0.49 (4)	4.84 ± 0.27 (8)	0.18	0.10
	*Excluding non compliant mothers*	4.09 ± 0.27 (7)	4.40 ± 0.68 (3)	4.84 ± 0.27 (8)	0.23	0.11
	**HDL cholesterol (mmol/l)**	1.40 ± 0.15 (7)	1.62 ± 0.10 (4)	1.45 ± 0.10 (8)	0.52^4^	0.67
	*With imputation*	1.40 ± 0.14 (8)	1.62 ± 0.10 (4)	1.45 ± 0.10 (8)	0.48^4^	0.75
	*Excluding non compliant mothers*	1.38 ± 0.17 (6)	1.69 ± 0.10 (3)	1.45 ± 0.10 (8)	0.37^4^	0.52
	**Triglycerides (mmol/l)^*3*^**	0.95 ± 0.15 (8)	0.86 ± 0.13 (4)	1.13 ± 0.16 (8)	0.51	0.23
	*Excluding non compliant mothers*	0.96 ± 0.18 (7)	0.80 ± 0.17 (3)	1.13 ± 0.16 (8)	0.51	0.13
Insulin like growth factors	**IGF1 (ng/ml)**	185.8 ± 12.4 (8)	195.8 ± 16.9 (4)	184.9 ± 12.8 (7)	0.87	0.33
	*With imputation*	185.8 ± 12.4 (8)	195.8 ± 16.9 (4)	185.7 ± 12.3 (8)	0.87	0.35
	*Excluding non compliant mothers*	178.1 ± 11.3 (7)	201.3 ± 22.6 (3)	184.9 ± 12.8 (7)	0.61	0.35
	**IGFBP3**	4119 ± 254 (8)	4268 ± 121 (4)	4798 ± 227 (7)	0.12	0.02^3^
	*With imputation*	4119 ± 254 (8)	4268 ± 121 (4)	4722 ± 232 (8)	0.14	0.05^3^
	*Excluding non compliant mothers*	4106 ± 293 (7)	4312 ± 160 (3)	4798 ± 227 (7)	0.17	0.06^3^
Adiposity	**Body Mass Index^*3*^**	22.6 ± 0.8 (8)	23.3 ± 1.3 (4)	23.4 ± 1.1 (8)	0.84	0.70
	*Excluding non compliant mothers*	22.5 ± 0.9 (7)	24.2 ± 1.4 (3)	23.4 ± 1.1 (8)	0.65	0.93
	**Dexa_Total_Fat (%)**	25.6 ± 2.8 (7)	35.7 ± 2.2 (4)	26.6 ± 2.4 (8)	0.06	0.12
	*With imputation*	25.3 ± 2.6 (8)	35.7 ± 2.2 (4)	26.6 ± 2.4 (8)	0.03	0.11
	*Excluding non compliant mothers*	25.6 ± 2.8 (7)	35.9 ± 3.0 (3)	26.6 ± 2.4 (8)	0.11	0.19
Urinary steroid ratios	**Urinary F/E ratio**	0.77 ± 0.07 (8)	0.81 ± 0.02 (2)	0.88 ± 0.08 (6)	0.53	0.53
	*With imputation*	0.77 ± 0.07 (8)	0.87 ± 0.07 (4)	0.88 ± 0.06 (8)	0.41	0.26
	*Excluding non compliant mothers*	0.78 ± 0.08 (7)	0.83 (1)	0.88 ± 0.08 (6)	0.65	0.57
	**Urinary THF + 5aTHF/THE ratio**	0.97 ± 0.09 (8)	0.88 ± 0.09 (2)	0.95 ± 0.18 (6)	0.92^4^	0.86
	*With imputation*	0.97 ± 0.09 (8)	0.82 ± 0.08 (4)	1.00 ± 0.15 (8)	0.65^4^	0.99
	*Excluding non compliant mothers*	0.96 ± 0.11 (7)	0.80 (1)	0.95 ± 0.18 (6)	0.87^4^	0.88
	**Testosterone – Males (nmol/l)^*3*^**	18.4 ± 1.5 (5)	15.8 (1)	21.2 ± 1.9 (5)	0.34	0.46
	*Excluding non compliant mothers*	19.3 ± 1.6 (4)	15.8 (1)	21.2 ± 1.9 (5)	0.42	0.46
	**Testosterone – Females (nmol/l)**	2.0 ± 0.2 (2)	2.1 ± 0.3 (3)	1.9 ± 0.3 (3)	0.85	0.57
	*With imputation*	1.9 ± 0.2 (3)	2.1 ± 0.3 (3)	1.9 ± 0.3 (3)	0.83	0.63^3^
	*Excluding non compliant mothers*	2.0 ± 0.2 (2)	2.4 ± 0.3 (2)	1.9 ± 0.3 (3)	0.57	0.84
	**SHBG – Males (nmol/l)^*3*^**	19.9 ± 3.6 (5)	18.3 (1)	18.1 ± 2.3 (5)	0.91	0.77
	*Excluding non compliant mothers*	19.4 ± 4.6 (4)	18.3 (1)	18.1 ± 2.3 (5)	0.96	0.77
	**SHBG – Females (nmol/l)^*3*^**	39.6 ± 15.5 (3)	41.0 ± 10.6 (3)	29.7 ± 12.8 (3)	0.16^4^	0.60
	*Excluding non compliant mothers*	39.6 ± 15.5 (3)	46.5 ± 15.9 (2)	29.7 ± 12.8 (3)	0.75	0.33

^1^ p value for difference between groups

^2^ p value for difference between groups adjusted for BMI, Dexa, age and gender (BMI or Dexa fat are omitted as covariates when they are the dependent)

^3^ Imputation not required.

^4^ p value has been derived by bootstrapping because residuals from ANOVA were not normally distributed (Shapiro-Wilk test found to be significant).

* Trial Group” refers to original experimental treatment allocation; **Standard error of the mean; ***Multiple imputation of missing values as described in text; ****Per protocol analysis excluding mothers with evidence for non-compliance as described in text

Table **6** shows between group differences in risk factors amongst offspring of mothers selected on the basis of nutritional status with the vitamins only group as the reference category. As with offspring of unselected mothers there is no strong evidence apparent of differences in individual risk factors between groups and no evidence of a pattern of lower risk in either of the energy supplementation groups compared to the vitamins only group. 

**Table 6:** Adult disease risk markers amongst offspring of mothers participating in the Sorrento studies of maternal nutritional supplementation who were assessed as adults: between group differences^1^ - Trial II (supplementation in nutritionally at risk mothers after 28 weeks).

**Risk marker**			**Protein, Carbohydrate and Vitamins**		**Carbohydrate and vitamins**	
			**Difference**	**(95% CI)**	**Difference**	**(95% CI)**
**Blood pressure**	**Mean 24hr Systolic BP (mmHg)**	Unadjusted	4.88	(-4.43 to 14.18)	0.75	(-10.37 to 11.87)
		Adjusted^*^	2.02	(-5.24 to 9.28)	1.97	(-7.10 to 11.03)
	**Mean 24hr Diastolic BP (mmHg)**	Unadjusted	0.75	(-6.48 to 7.98)	-3.50	(-12.14 to 5.14)
		Adjusted	1.10	(-6.32 to 8.52)	-5.21	(-14.48 to 4.05)
**Markers of glucose tolerance and insulin resistance**	**Fasting glucose (mmol/l)**	Unadjusted**^*2*^**	-0.01	(-0.31 to 0.29)	-0.52	(-0.89 to -0.15)
		Adjusted	-0.11	(-0.51 to 0.28)	-0.46	(-0.98 to 0.05)
	**30 mins post load glucose (mmol/l)**	Unadjusted	-1.41	(-3.08 to 0.25)	-1.71	(-3.75 to 0.32)
		Adjusted	-1.73	(-3.81 to 0.36)	-1.48	(-4.20 to 1.25)
	**120 mins post load glucose (mmol/l)**	Unadjusted	0.76	(-0.65 to 2.17)	0.25	(-1.48 to 1.98)
		Adjusted	0.77	(-0.92 to 2.47)	-0.86	(-3.08 to 1.35)
	**Fasting insulin (pmol/l)**	Unadjusted	-23.70	(-57.69 to 10.29)	-35.50	( -96.12 to 25.12)
		Adjusted	-18.35	(-59.92 to 23.22)	-54.46	(-126.73 to 17.82)
	**30 mins post load insulin (pmol/l)**	Unadjusted**^*2*^**	-132.58	(-431.82 to 166.67)	-66.06	(-419.03 to 286.90)
		Adjusted	4.33	(-423.76 to 432.41)	27.12	(-402.81 to 457.05)
	**120 mins post load insulin (pmol/l)**	Unadjusted**^*2*^**	-25.18	(-431.19 to 380.84)	-173.99	(-585.93 to 237.95)
		Adjusted	50.75	(-480.03 to 581.53)	-656.50	(-1584.18 to 271.19)
	**HbA1c (%)**	Unadjusted	-0.11	(-0.44 to 0.21)	-0.14	(-0.54 to 0.26)
		Adjusted	-0.07	(-0.35 to 0.21)	0.07	(-0.29 to 0.44)
	**Cpeptide fasting (nmol/l)**	Unadjusted	-0.21	(-0.48 to 0.06)	-0.24	(-0.57 to 0.09)
		Adjusted	-0.14	(-0.31 to 0.04)	-0.27	(-0.50 to -0.04)
	**B cell function (%)**	Unadjusted	-33.04	(-79.16 to 13.07)	-40.48	(-122.75 to 41.78)
		Adjusted	-22.60	(-73.35 to 28.15)	-63.98	(-152.21 to 24.26)
	**Insulin sensitivity (%)**	Unadjusted**^*2*^**	204.16	(-3.75 to 412.08)	164.84	(-49.78 to 379.46)
		Adjusted	128.04	(-211.42 to 467.50)	379.62	(-210.61 to 969.85)
	**Insulin resistance**	Unadjusted	-0.44	(-1.06 to 0.18)	-0.66	(-1.77 to 0.45)
		Adjusted	-0.34	(-1.11 to 0.42)	-1.02	(-2.35 to 0.31)
**Serum lipids**	**Total cholesterol (mmol/l)**	Unadjusted	-0.73	(-1.53 to 0.08)	-0.54	(-1.52 to 0.45)
		Adjusted	-1.01	(-1.94 to -0.08)	-0.67	(-1.89 to 0.54)
	**HDL cholesterol (mmol/l)**	Unadjusted**^*2*^**	-0.06	(-0.36 to 0.25)	0.16	(-0.21 to 0.53)
		Adjusted	-0.16	(-0.60 to 0.28)	0.03	(-0.55 to 0.60)
	**Triglycerides (mmol/l)**	Unadjusted	-0.19	(-0.63 to 0.25)	-0.28	(-0.81 to 0.26)
		Adjusted	-0.11	(-0.60 to 0.39)	-0.54	(-1.18 to 0.11)
**Insulin like growth factors**	**IGF1 (ng/ml)**	Unadjusted	0.89	(-36.89 to 38.68)	10.89	(-34.87 to 56.65)
		Adjusted	16.75	(-24.06 to 57.57)	35.13	(-14.69 to 84.94)
	**IGFBP3**	Unadjusted	-678.88	(-1347.79 to -9.96)	-529.75	(-1339.85 to 280.35)
		Adjusted**^*2*^**	-521.78	(-1080.40 to 36.83)	-994.69	(-1646.08 to -343.31)
**Adiposity**	**Body Mass index**	Unadjusted	-0.76	(-3.60 to 2.08)	-0.09	(-3.57 to 3.38)
		Adjusted	-0.06	(-3.24 to 3.12)	-1.52	(-5.56 to 2.53)
	**Dexa total fat (%)**	Unadjusted	-1.02	(-8.32 to 6.29)	9.06	( 0.42 to 17.71)
		Adjusted	-1.23	(-6.76 to 4.29)	5.21	(-1.37 to 11.79)
**Urinary steroid ratios**	**Urinary F/E ratio**	Unadjusted	-0.11	(-0.32 to 0.10)	-0.07	(-0.39 to 0.25)
		Adjusted	-0.14	(-0.42 to 0.14)	-0.04	(-0.53 to 0.45)
	**Urinary THF + 5aTHF/THE ratio**	Unadjusted**^*2*^**	0.03	(-0.29 to 0.34)	-0.05	(-0.47 to 0.37)
		Adjusted	0.02	(-0.41 to 0.45)	0.19	(-0.57 to 0.94)
	**Testosterone – Males (nmol/l)**	Unadjusted	-2.80	( -8.26 to 2.66)	-5.44	(-14.89 to 4.01)
		Adjusted	-4.46	(-13.73 to 4.81)	-2.26	(-13.08 to 8.55)
	**Testosterone – Females (nmol/l)**	Unadjusted	0.13	(-1.04 to1.31)	0.23	(-0.81 to 1.28)
		Adjusted	-0.77	(-4.43 to 2.89)	1.47	(-5.57 to 8.50)
	**SHBG – Males (nmol/l)**	Unadjusted	1.82	( -7.98 to 11.62)	0.20	(-16.77 to 17.17)
		Adjusted	-4.57	(-25.39 to 16.25)	-4.65	(-28.95 to 19.65)
	**SHBG – Females (nmol/l)**	Unadjusted**^*2*^**	9.29	(-21.00 to 39.58)	10.39	(-19.48 to 40.27)
		Adjusted	-0.62	(-113.65 to 112.41)	32.80	(-77.55 to 143.15)

^1^ between group differences are based on the complete case analysis and are shown with Vitamins only group as reference category

^2^ estimates have been bootstrapped as residuals were not normally distributed (Shapiro-Wilk test <0.05)

* adjusted for BMI, Dexa, age and gender (BMI or Dexa fat are omitted as covariates when they are the dependent)

## Discussion

Amongst young adult offspring of mothers randomised to receive one of three nutritional supplementation regimes during pregnancy either unselected or selected on the basis of evidence of their nutritional status we found no evidence that supplementation influenced adult risk markers of cardiovascular disease, diabetes or cancer. Amongst mothers with evidence of under-nutrition, protein-energy supplementation during the final pregnancy trimester was associated with increased birth weight but this increase was not associated with any strong or consistent change in any marker of adult disease risk measured. These risk markers included many of those found to be associated with birth size in observational studies such as blood pressure [27], markers of glucose tolerance and insulin resistance [28]; blood lipids and adiposity along with more novel markers of cardiovascular risk based on steroid hormones and possible markers of cancer risk based on insulin like growth factors. It has been suggested that adult effects of fetal nutrition may vary according to adult adiposity[29]. Adjustment for adult BMI and total body fat did not change our results.

### Comparison with other evidence

Three other trials of the effectiveness of nutritional supplementation during pregnancy on perinatal and early childhood outcomes have now been extended to include later childhood or early adult follow up[11-14]. These trials have all been undertaken in low or middle-income countries using different protein-energy supplements amongst mothers unselected in relation to nutritional status. Follow up of offspring of participants in these trials has found no evidence of effects on blood pressure or adiposity in adolescence or early adulthood[11-14]. In one trial (which was non-randomised) there was evidence of a favourable effect on fasting insulin and insulin resistance as estimated using the HOMA model[14]. We did not find evidence of this effect. In another study weak evidence of a favourable effect on blood lipids (higher HDL cholesterol and lower triglycerides) was found[13]. Again we found no evidence of such an effect.

Many of the risk markers we assessed were not measured in these follow up studies so direct comparison is not possible. We chose measures that appeared to provide the most valid index of the risk parameter of interest in relation to all the elements and mechanisms of risk previously discussed in relation to the fetal origins hypothesis. Thus in addition to standard clinic assessments we used 24 hour ambulatory blood pressure measurement and we assessed body composition and adiposity through DEXA scanning in addition to standard measures of height and weight. Fetal corticosteroid exposure has been suggested as an alternative mechanism whereby smaller birth size could be associated with higher risk of later cardiovascular disease principally through an effect on hypertension or adiposity[30]. Because of this in addition to measures of blood pressure and adiposity we examined urinary markers of the aspects of steroid metabolism mediating these possible effects. Again we found no evidence of an association with nutritional supplementation. Because of evidence that fetal over-nutrition may increase later risk of breast cancer, childhood leukaemias and some other malignancies we felt it important to also consider risk markers of cancer in our study[20]. We found no association between supplementation and higher levels of insulin like growth factors. Other evidence suggests that higher infant milk protein intake is associated with lower levels of IGF1 in later life[31-33]. We found very weak evidence of lower IGF1 in offspring of unselected mothers receiving protein energy supplements and slightly stronger evidence of lower IGFBP3 in offspring of undernourished mothers who received these supplements. 

### Strengths and limitations

The main strength of this study is its experimental design. Also, our study was undertaken in a high income setting with facilities for assessment of a wide range of risk markers using relatively sophisticated technologies. We also provided evidence in relation to the value of targeted supplementation based on nutritional assessment compared to universal supplementation. Finally this study provided evidence in relation to a supplementation intervention feasibly delivered within a health care system typical of high-income countries. The main limitation of this study is its small size and consequently limited power and precision. Sample size was set by the original Sorrento study and was further reduced by incomplete follow up. These considerations are particularly relevant to the second Sorrento trial amongst mothers selected on the basis of their evidence of under-nutrition. Considering the age of the study population and the fact that assessments involved a clinic visit, follow up rates of 50% amongst those with valid contact details were relatively high. Amongst individuals with incomplete assessment data we used multiple imputation of missing values which increased power and did not suggest any important bias in the complete case analysis. Our participants were all of South Asian ethnicity however observational evidence supporting the “fetal origins” hypothesis has also been reported from South Asian populations[34]. Given the disproportionate experience of cardiovascular and metabolic disease amongst South Asians and the apparent failure of conventional risk factors to fully explain this increase, it is arguably important to study novel risk factors in this population[35]. Nutritional supplements were delivered to unselected study mothers from the second pregnancy trimester and to mothers selected because of evidence of under-nutrition from the third pregnancy trimester. We were thus unable to investigate the possible influence of nutrition earlier in pregnancy on offspring disease risk. In two of the other follow up studies discussed above supplements were delivered to pregnant women identified on the basis of missed menstruation thus supplementation typically started in the first trimester[12-14]. In the other trial, supplementation commenced in the second trimester[11]. It has been suggested that it is the rapid postnatal “catch –up” growth of the growth retarded fetus rather than fetal growth retardation per se that is the important influence on adult disease risk[36]. As we had no information on growth in the first year of life we could not examine this hypothesis. Follow up amongst adult offspring was only 50% and it is possible that individuals who were assessed as adults were different from those who were not, in terms of characteristics also associated with disease risk such as social position. We had no individual level measures of adult social position available on non-responders to allow us to investigate this question. The fact that individuals who were assessed as adults were relatively healthy (table 1) may have reflected the fact that they were also relatively socially advantaged however any selection bias associated with this is unlikely to have distorted associations between trial allocation and adult disease risk markers[37].

### Conclusions and implications

We found no evidence that, irrespective of other benefits it may have, improving the nutritional status of pregnant women is likely to have substantial long term effects on offspring risk of cardiovascular disease or diabetes. We also found no evidence of any substantial adverse effects on increased cancer risk. It is important to note that we lacked power to detect small effects in relation to these outcomes. Within this caveat our study suggests that rather than expanding public health policy around cardiovascular prevention to include fetal nutrition preventive policy should continue to be focused on the established adult behavioural and physiological risk factors hypertension, dyslipidaemia, diabetes, obesity, lack of exercise and smoking alongside the social disadvantage that reinforces adverse risk profiles in these. 

## Supporting Information

Figure S1 **Flow Diagrams showing maternal participation in original Sorrento trial and offspring follow up.**
(DOCX)
