# Supplementary material for: Effects of Nutritional Supplementation during Pregnancy on Early Adult Disease Risk: Follow Up of Offspring of Participants in a Randomised Controlled Trial Investigating Effects of Supplementation on Infant Birth Weight
Source: PLoS One. 2013 Dec 13;8(12):e83371. doi: 10.1371/journal.pone.0083371 (PMC3862758; doi:10.1371/journal.pone.0083371)
Supplement: Figure S1 — Flow Diagrams showing maternal participation in original Sorrento trial and offspring follow up. (DOCX) [file pone.0083371.s001.docx]

**Figure S1**

**Trial I: Supplementation in all mothers from 18 weeks**

## Mothers enrollment

All mothers booking at Sorrento April ’79 – October ’79 (n=153)

Randomized (n=153)

## Mothers allocation and inclusion in original analysis*

Allocated to vitamins only (n= 45)

Included in original analysis (n=45)

Allocated to energy and vitamins supplements (n= 57)

Included in original analysis (n=50)

Moved away n=3, miscarriage n=1, perinatal death n=2, twin pregnancy n=1

Allocated to protein, energy and vitamins supplements (n= 51)

Included in original analysis (n=47)

Moved away n=1, miscarriage n=1, perinatal death n=1, twin pregnancy n=1

## Offspring traced and invited for assessment**

n=41

n=40

n=38

**Trial II: Supplementation in nutritionally at risk mothers after 28 weeks**

## Offspring assessed and included in current analysis**

n=21

n=23

n=21

## Mothers enrollment

All mothers booking at Sorrento November ’79 – June ’80 (n=130)

Not randomized as appeared adequately nourished n=85

Randomized (n=45)

## Mothers allocation and inclusion in original analysis*

Allocated to vitamins only (n= 14)

Allocated to energy and vitamins supplements (n= 17)

Allocated to protein, energy and vitamins supplements (n= 14)

## Offspring traced and invited for assessment**

n=13

n=14

n=15

## Offspring assessed and included in analysis**

N=8

N=8

N=4

*****Analysis is by intention to treat; compliance was checked in all mothers by study staff and receipt of empty containers confirmed

**Of 283 mothers 275 were traced as living and registered with a UK GP, 7 GPs declined to provide maternal contact details, 268 mothers were contacted and provided contact details for 236 offspring (10 mothers declined to provide offspring contact details, 13 reported offspring living abroad, 4 reported offspring deceased, 5 did not respond).

***All traced offspring (n=236) were contacted, 118 attended for assessment and 118 declined participation, 33 individuals assessed are not considered in these analyses as their mothers participated in Trial II but showed no evidence of nutritional risk at 28 weeks
